# Periodontoclasia*Extracts from a paper read before the Pennsylvania State Society and published in full in *Cosmos*, January, 1922.

**Published:** 1922-03

**Authors:** John O. M’Call


### PERIODONTOCLASIA*

BY JOHN O. M'CALL, D.D.S.

Periodontoclasia is a disease producing or tending to produce a breaking down of any or all of these tissues. Since there are three distinct tissues in this group, and since disease may primarily attack any one of these tissues with but minor influence on the others, it follows that we have at least three types of periodontal disease. As a matter of fact, it is found more convenient to differentiate five types. And while it is a common occurrence to find two or more of these types blended in a complex or fusion type, yet it is by no means rare to find cases presenting pure examples of each of the five types. They are as follows:

1. *Recession.*—Wasting away of the marginal gingiva, together with the crest of the alveolar process, resulting in exposure of the cementum.

2. *Gingivitis.*—Inflammation of the gingival tissue. There are several sub-types of gingivitis, among which is the so-called "trench mouth."

3. *Alveoloclasia.*—Wasting away of the alveolar process, especially the inner wall of the alveolus, whereby the tooth becomes movable in its socket.

4. *Pericementoclasia.*—Dissolution of the fibers of the pericementum and of the alveolar process, without destruction of the overlying gingival tissue. It results in the formation of a pus pocket and constitutes, if any type does, the so-called pyorrhea alveolaris.

5. *Periodontal abscess.*—Any infection of the periodontium whose inflammatory exudate drains elsewhere than through the gingival crevice. This type of periodontal disease is seen to be rather sharply differentiated

---

* Extracts from a paper read before the Pennsylvania State Society and published in full in *Cosmos*, January, 1922.

from the diseases commonly spoken of as having their inception at the gingival margin. It includes the various infections which arise from pulp-canal infection. It also includes pericemental abscess, which seems to arise neither at the gingival margin nor the tooth apex, and gingival abscess. Gingival abscess is the result of an infection introduced into the gingival crevice. It may occur without previous periodontal disease having been established or it may occur in the soft tissue over a previously formed pocket. In either event the gingival abscess exhibits a definite tendency to drain through the external surface of the cemental or alveolar gingiva rather than through the gingival crevice.

Traumatic occlusion may occur in mouths containing thirty-two sound teeth in apparently perfect alignment. It is practically always present in mouths in which extraction of any teeth, except the third molars, has taken place, unless the space so created has been quickly filled with an artificial substitute. It is common in mouths having extensive restorations, whether large fillings, crowns, fixed or removable bridges or dentures. In a word, traumatic occlusion is to be found in varying degree in nearly every adult mouth, and is extremely common in the mouths of the young.

IN THE MANAGEMENT OF PERIODONTOCLÀSIA

As nearly as it is possible to lay out a routine procedure, the management of a case of periodontoclasia may be summed up in the following steps:

1. Diagnosis—determining the type and extent of disease.

2. Determination of etiology.

3. Determination, as nearly as possible, of prognosis.

4. Relief and removal of etiological factors in order of importance.

5. Maintenance of cleanliness, correct occlusion and tissue tone.

### REMEDIAL PROCEDURES

Before considering other types of periodontal disease it may be well to sum up the remedial procedures proposed for the average case of pericementoclasia, that is, one involving pocket formation, in the proper sequence:

*First.*—Correction of traumatic occlusion, usually by grinding of the offending cusps or other occluding surfaces. Unless the mouth is conspicuously neglected, no scaling is done at the first visit. If the deposits are so gross as to make it evident that no response is to be expected until they are removed, scaling will be done at this sitting, but is to be carried no farther than one millimeter beneath the existing margin of the gingiva. At subsequent sittings, the mouth is examined for improvement in coloration and firmness of teeth if they have been loose.

*Second.*—Institution of a correct method of using the toothbrush, so that stimulation of gingival circulation is begun. If there are deep pockets on buccal or lingual root surfaces, they are explored to determine the character of the deposits. If these deposits are nodular or perceptibly rough, they are dressed down, but not completely removed. The object of this is to avoid the brushing and massaging of the overlying gum against a rough surface such as was previously presented in these pockets.

*Third.*—Steps are taken to restore approximal support by restoration of contact points, replacement of lost teeth, etc. If fixed bridgework is indicated, it is not to be placed until the final scaling of abutment teeth is completed. But fillings, inlays and removable restorations are usually to be placed before final scaling of the root surfaces, unless it is apparent that changes in the contour of the soft tissues may occur which will alter the adaptation of appliances constructed before the scaling is done.

*Fourth.*—Correction of unfavorable systemic conditions, if needed, is begun by the physician. The prescription of diet based on the requirements as revealed in blood chemistry is a recent development which bids fair to be an important part of treatment.

*Fifth.*—The case has been carefully observed as to evidences of improvement in the color of the gingivæ, etc. It will usually be found that coincidentally with the lightening in color, there is a diminution in the flow of pus, and a perceptible tightening of loose teeth. There has been no medication, no vaccine. In cases exhibiting considerable flabbiness of the marginal gingivæ, an astringent mouthwash may be employed with benefit. The final scaling or curettage of the denuded root surface is now undertaken. And here, as in other steps, only a 100 per cent. operation will give even passable results. A 75 per cent. filling, or even a 50 per cent. filling may serve the purpose of a better one, differing mainly in that its life is shorter. But in the surgery of the root surface, it is all or nothing. There is no place for the 75 per cent. operation.

*Sixth.*—The mouth is kept clean both by the dentist and the patient. If pockets do not heal up, the case is reviewed to discover any possible errors in diagnosis, prognosis, or previous treatment. If these points are satisfactory, the root surface and its overlying gingival tissue are kept clean and fresh by delicate currettage, until the desired result is obtained or the case is decided to have an unfavorable prognosis as regards the attachment of that tissue. In the latter case the patient is instructed to return at more frequent intervals for prophylactic treatment.

With the systematizing of etiology and classifying of the types of dental periclasia, their practical application in treatment becomes a fairly simple and logical procedure. It is thus possible to give up the empirical treatment of symptoms and to direct our attention to the elimination of causes. Needless to say, when diagnosis of the type of disease and its etiology are correct, and correct treatment is given, cases having a favorable prognosis give results which make this branch of dental practice a pleasure rather than a travail.

